# Advancements in Polyethylene Oxide (PEO)–Active Filler Composite Polymer Electrolytes for Lithium-Ion Batteries: A Comprehensive Review and Prospects

**DOI:** 10.3390/ma17174344

**Published:** 2024-09-02

**Authors:** Hazlina Junoh, Nuha Awang, Hazirah Syahirah Zakria, Nurul Amira Shazwani Zainuddin, Nik Abdul Hadi Md Nordin, Nuor Sariyan Suhaimin, Tomoya Enoki, Takahiro Uno, Masataka Kubo

**Affiliations:** 1 Faculty of Engineering, Department of Applied Chemistry, Mie University, 1577 Kurimamachiyacho, Tsu 514-8507, Japan; hazlina.eng@mie-u.ac.jp (H.J.); hazirah.eng@mie-u.ac.jp (H.S.Z.); 422de02@m.mie-u.ac.jp (N.A.S.Z.); 424d001@m.mie-u.ac.jp (T.E.); uno@chem.mie-u.ac.jp (T.U.); 2 Plant Engineering Technology (PETech), Malaysia Institute of Industrial Technology (UniKL, MITEC), Universiti Kuala Lumpur, Jln Persiaran Sinaran Ilmu, Bandar Seri Alam, Masai 81750, Malaysia; nuha.awang@unikl.edu.my; 3 Department of Chemical Engineering, Universiti Teknologi PETRONAS, Seri Iskandar 32610, Malaysia; nahadi.sapiaa@utp.edu.my; 4 Advanced Membrane Technology Research Centre (AMTEC), School of Chemical and Energy Engineering, Faculty of Engineering, Universiti Teknologi Malaysia, Skudai 81310, Malaysia; sariyan@utm.my

**Keywords:** polyethylene oxide, lithium−ion batteries, active fillers, composite polymer, electrolyte, lithium-ion

## Abstract

Polyethylene oxide (PEO) has become a highly sought−after polymer electrolyte for lithium−ion batteries (LIBs) due to its high ionic conductivity, strong mechanical properties, and broad electrochemical stability range. However, its usefulness is hindered by its limited ionic conductivity at typical temperatures (<60 °C). Many researchers have delved into the integration of active fillers into the PEO matrix to improve the ionic conductivity and overall efficiency of composite polymer electrolytes (CPEs) for LIBs. This review delves deeply into the latest developments and insights in CPEs for LIBs, focusing on the role of PEO–active filler composites. It explores the impact of different types and morphologies of active fillers on the electrochemical behavior of CPEs. Additionally, it explores the mechanisms that contribute to the improved ionic conductivity and Li−ion transport in PEO−based CPEs. This paper also emphasizes the present obstacles and prospects in the advancement of CPEs containing PEO–active filler composites for LIBs. It serves as a valuable reference for scientists and engineers engaged in the domain of advanced energy storage systems, offering insights for the forthcoming development and enhancement of CPEs to achieve superior performance in LIBs.

## 1. Introduction

As the world continues to advance industrially and technologically, the need for energy is increasing. However, traditional energy systems face challenges such as the intermittent nature of renewable sources when demand is high. Moreover, fluctuations in energy demand create challenges in maintaining a stable and reliable energy supply. Balancing the supply and demand becomes more complex when demand peaks do not align with the periods of maximum renewable energy production [1]. These challenges highlight the need for effective energy storage solutions to bridge the gaps, ensuring a reliable and resilient energy system. LIBs have emerged as crucial competitors in pursuing efficient and sustainable energy storage solutions, powering various applications across portable electronics, electric vehicles, and grid-scale energy storage systems [2]. Lithium batteries have a long history, but it was after Harris’s research in early 1958 that the development of LIBs gained significant interest. Harris conducted an experimental study on the solubility of lithium with various electrolytes and materials. His study discovered that lithium-ion batteries were stable due to a passivation layer that had formed, which prevented the electrolytes from reacting with lithium while still allowing ionic transport [3,4]. Stanley Whittingham later initiated the “self−recharging” battery during the 1970s, aiming to develop a faster−recharging battery for a future powered by renewable energy without using fossil fuel. The prototype of the modern LIBs, which uses carbonaceous anodes rather than lithium metal, was developed in 1985 by Akira Yoshino and commercially introduced in the early 1990s [5,6].

Despite their widespread use, conventional liquid electrolytes composed of organic solvents and lithium salts suffer from several drawbacks that curb LIB performance and safety. Thus, these limitations have spurred intensive research into alternative solid−state electrolytes (SSEs) [7]. Among the alternatives, polymer electrolytes have garnered significant attention for their potential to encounter the shortcomings of liquid electrolytes. Furthermore, polymer electrolytes can be engineered to possess high ionic conductivity by facilitating efficient ion transport within battery systems. These distinguished properties make polymer electrolytes highly suitable for applications in lithium-ion batteries for portable electronics, electric vehicles, and grid−scale energy storage.

PEO is a solid polymer electrolyte with a flexible chain structure. The ether oxygen (EO) atoms in the ethylene oxide chain can interact with cations such as Li^+^, providing a strong dissociating property for a wide variety of salts. Additionally, the excellent chain flexibility and segmental motion of EO segments facilitate Li^+^ conduction. However, at room temperature, the crystalline aggregation of PEO restricts Li^+^ conduction. Overcoming the limitation of electrolytes, CPEs perfectly combine the advantages of inorganic solid electrolytes (ISEs) and solid polymer electrolytes (SPEs) to provide a wider range of benefits. While the high ionic conductivity and mechanical strength of ISEs are retained, CPEs also offer good flexibility, improved interfacial conditions, and excellent processing properties of SPEs [8]. There are many reported methods for the preparation and design of CPEs, such as polymer blending, crosslinking polymer matrices, comb-branched copolymers, etc.; however, the current practices are commonly the solution casting method, the electrostatic spinning method, and in situ polymerization [9,10].

The ion transport within CPEs is primarily influenced by the polymer chains. Therefore, it is important to consider the size, concentration, and hybridization strategies of fillers in order to synthesize high-performance CPEs [11]. Integrating inorganic fillers into PEO matrices to develop CPEs has the potential to synergistically boost the electrochemical performance of PEO−based electrolytes by promoting ionic conductivity (by lowering polymer crystallinity or/and creating additional ionic pathways), enhancing mechanical strength, and stability [2]. The fillers incorporated can be further subdivided into active and passive fillers. However, active fillers are known to dominate over passive fillers in CPEs for lithium-ion batteries because they directly address the critical need for high ionic conductivity and electrochemical stability. Meanwhile, passive fillers play a supportive role in enhancing mechanical and thermal properties; the primary advancements necessary for the efficient and effective operation of lithium-ion batteries are driven by the inclusion of active fillers. Thus, the focus on active fillers is critical for developing high-performance CPEs that can meet the demanding requirements of advanced lithium-ion batteries [12]. Reviewing current developments in PEO–active filler CPEs for LIBs and offering predictions for their future are the goals of this extensive study. This study aims to clarify the principles behind the improved features and possible uses of these composite electrolytes in next−generation energy storage devices by examining their synthesis processes, characterization strategies, and electrochemical performance.

## 2. Conventional LIBs

The LIB is a rechargeable electrochemical energy storage technology that has become the primary power source for a wide range of applications, including portable electronics, electric vehicles, and renewable energy systems. The basic components of LIBs consist mainly of an anode, cathode, electrolyte, and separator, as well as the external circuit for delivering power. LIBs can undergo two different mechanisms, called charge and discharge mechanisms. During the charging mechanism, the external power source is applied, triggering the movement of lithium ions (Li^+^ ions) from the cathode to the anode side while passing through the electrolyte and separator. While discharging, the released Li^+^ ions from graphite (anode) pass through the electrolyte and separator, and then diffuse into the delithiated cathode. The discharging of the battery indicates that the energy stored in the anode is being released to power up the load (electronic devices or electric vehicles). In this system, both electrodes undergo the redox reaction during the charging/discharging process [13], which can be dictated as follows:

- Charging process
  - Oxidation reaction: (Anode) LiMO_2_ → Li_1-x_MO_2_ + _x_Li^+^ + _x_e^−^
  - Reduction reaction: (Cathode) _y_C + _x_Li^+^ + _x_e^−^ → Li_x_C_y_
- Discharging process
  - Oxidation reaction: (Anode) Li_x_C_y_ → _y_C + _x_Li^+^ + _x_e^−^
  - Reduction reaction: (Cathode) Li_1-x_MO_2_ + _x_Li^+^ + _x_e^−^ → LiMO_2_

**Figure 1:**
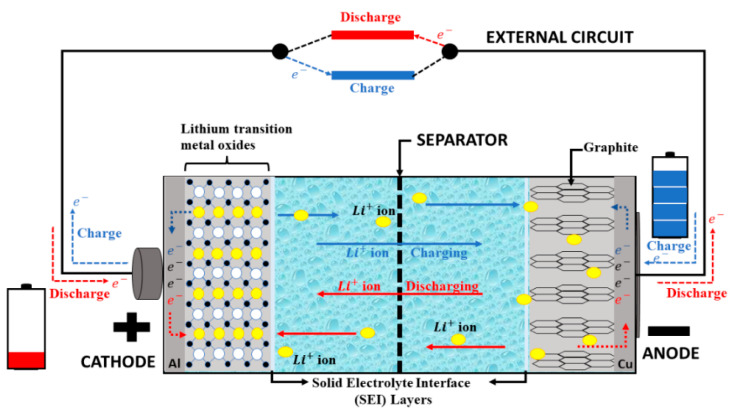
Schematic diagram of the charging/discharging process for the Li^+^ ion cell [17,18].

Basically, the cathode (positive terminal) allows the lithium ions to be inserted or removed reversely and typically made from lithium transition metal oxide−based materials (lithium iron phosphate (LiFePO_4_), lithium cobalt oxide (LiCoO_2_), or lithium manganese oxide (LiMn_2_O_4_)). Inversely, the anode (negative terminal) serves as the negative electrode where the lithium−ion will be stored [14,15] and is mainly made of carbon, specifically graphite. Conventional LIBs consist of a liquid electrolyte that stands in between the electrodes and allows the lithium ions to easily travel between the electrodes. The electrolyte should possess high electronic insulating properties as well as high lithium ionic conductivity. A solution of lithium salt (mainly lithium hexafluorophosphate (LiPF_6_)) with a mixed-organic solvent (ethylene carbonate (EC) or dimethyl carbonate (DMC)) is usually used as the electrolyte with a conductivity of 100 × 10⁻^4^ S cm^−1^ [16]. Figure 1 illustrates the conventional LIB, which is basically composed of a thin sheet of microperforated plastic that acts as a separator, preventing the direct contact of electrodes, which leads to a short circuit while simultaneously allowing the ions to transfer [17,18]. The current collector is used to wrap the anode and cathode materials, which is mainly made out of copper or aluminum foils, respectively [17,18].

Although commercial LIBs possess sophisticated features such as a rechargeable device, high ionic conductivity, and excellent wettability, they also face some drawbacks due to the carbonate-based liquid electrolyte. This contributes to several issues, such as (1) safety issues while using the highly flammable and volatile organic solvents due to leakage, (2) parasitic interface reactions due to the growth of lithium dendrites on the surface of the anode, and (3) unexpected increases in temperature due to the internal short circuit. These subsequent issues will eventually bring about catastrophic consequences, such as incidences of burning and explosion of Li-ion batteries [19]. Thus, to prevent all of the issues while maintaining the specialty of rechargeable LIBs, high ionic conductivity, and excellent wettability, some research has been conducted to replace liquid electrolytes with solid-state electrolytes.

## 3. Solid-State Electrolytes (SSEs)

The bottleneck in the development of SSEs is mainly due to the focus on delivering high conductivity, free-dendrite formation, high mechanical and thermal stability, long-term stability, high life cycles, compactness, and safe handling to be used in electrical energy storage (EES) technologies [20,21]. SSEs can provide a higher energy density for the batteries due to their wider electrochemical window and better compatibility with lithium metal anodes [22]. Apart from the special advantages of SSEs, more research and development (R&D) is required in order to overcome the associated challenges, such as (1) the penetration of metallic lithium into solid materials, (2) the stabilization interface between electrodes and the electrolyte, and (3) maintaining physical contact between electrodes and the electrolyte [23]. SSEs can be classified into two large categories, which are (1) quasi−solid−state electrolytes (QSSEs) and (2) all solid−state electrolytes (ASSEs). QSSEs basically consist of a liquid component (solvent or plasticizer) and a solid matrix, which, respectively, provide a permeating medium for ion transportation and mechanical stability to the system [24]. Gel polymer electrolytes (GPEs), ion gel electrolytes, and gel electrolytes are the major classes of QSSEs. Despite the sophisticated structure, QSSEs are impracticable since they can lead to feeble mechanical and structural stability of the electrolyte [22]. Meanwhile, ASSEs are composed exclusively of a solid matrix and may be further classified into three types: (1) ISEs, (2) SPEs, and (3) CPEs [24].

For the past few centuries, active development has been carried out on the fabrication of ISEs and SPEs (Table 1), respectively, with respect to the first discovery 200 years ago of fast solid−state ion transportation in lead fluoride (PbF_2_) and silver sulfide (Ag_2_S) electrochemical cells by Michael Faraday [20]. Moreover, Shalably et al. [21] and Yang et al. [25] have provided comprehensive reviews of the different types of electrolyte materials for SSEs that have burgeoned in the past few years.

ISEs, which are essentially composed of oxides and sulfides, have garnered significant attention for their higher ionic conductivity and electrochemical stability window. However, ISEs suffer from fragility and discontinuous contact with the electrode, which hampers Li−ion transportation. For instance, the oxide solid electrolytes require a high sintering temperature (>1000 ℃) with high rigidity, resulting in discontinuity of contact with the electrode and the inherent brittleness of the materials. Additionally, the sulfide solid electrolytes possess limited thermodynamic stability and are very sensitive to water (H_2_O) and oxygen (O_2_), which can easily react with air moisture to release extremely hazardous gaseous hydrogen sulfide (H_2_S) [25]. Therefore, a flexible structure of SPEs was introduced for creating continuous contact with the electrode as well as a resilient structure of electrolytes.

Generally, SPEs consist of hundreds of repeating units’ monomers with a dissolved metal salt to form a conductive polymer matrix [26]. Low lattice energies are induced between the donor atoms from the polymer matrix and the Li cation, which eventually makes the whole system conductive [27]. The first concept of SPEs was introduced in the 1970s by Fenton et al. [13] and Wright [14], whereby they discovered the ionic conductivity of the complex PEO and alkali metal ions, which were eventually implemented in LIB applications. The pivotal components of lithium batteries have been thoroughly studied by Professor Michel Armand with his first proposal of a lithium−ion conducting polymer electrolyte for all−solid−state lithium batteries in 1978 [28]. Subsequently, in 1986, the sulfonamide family based on delocalized anions was developed by Professor Michel Armand and team, and the advancement of SPEs has continued until today due to their high strength, low heat transfer, good insulation, and resistance to corrosion; moreover, polymer materials have been utilized in many aspects of our daily lives [26]. Additionally, polymers are relatively easy to synthesize, with low mass density, superior processability, safety, flexibility, and high stability, and they are lightweight, highly compact, easy to recycle, cost−effective, and possess good mechanical properties [28,29], making SPEs superior to ISEs for LIBs.

As they gain high interest in the production of LIBs, the thermal stability of SPEs becomes a major concern. The thermal stability of SPEs consists of a few important parameters, which are (1) glass transition temperature (T_g_), (2) initial decomposition temperature (I_t_), and (3) tensile strength (T_s_) [29]. As the temperature changes, it subsequently affects the stiffness and ionic conductivity of the SPEs. Thus, it is crucial to pay attention to the T_g_ of the materials. For instance, above the T_g_ value, the polymer typically undergoes state changes from glass to a high−elastic state with respect to the critical temperature and transition process of materials. Additionally, it is worth noting that when the mechanical properties and T_s_ of SPEs are below the required value, the growth of lithium dendrite can occur, which eventually leads to thermal runaway [29]. Furthermore, the value of T_g_ also contributes to the movement of polymer chains in SPEs. The movement of polymer chains is directly correlated with the mechanism of ion transportation within the polymer matrix. Polymers with a low T_g_ value are preferable to use since the segmental movement of polymer chains only happens at temperatures above the T_g_ value [27].

The free electron pairs of heteroatoms (oxygen or nitrogen) in the polymer chain form complexes with the metal ions and subsequently help the movement of metal ions between the coordination sites [26]. The other type of ion transportation mechanism in SPEs is the ion hopping process. In the latter mechanism, the metal ions and polymer chains overcome the energy barrier by breaking bonds that allow the metal ions to hop to the adjacent polymer chains [26]. Moreover, depending on the value of the molecular weight (M_w_), PEO is also known as PEG. Basically, at a higher M_w_ value above 20 kg/mol, the compound is classified as PEO. Whereas at an M_w_ value lower than 20 kg/mol, the compound is called PEG. Table 2 summarizes the properties of PEO at different M_w_ values. Basically, ideal PEOs possess low M_w_ and T_g_ values, respectively. However, these PEOs resulted in limited ionic conductivity at room temperature (10^−9^ to 10^−6^ S cm^−1^), a narrow electrochemical window, poor electrode and electrolyte interface compatibility [30], and low thermal and mechanical strength, which hindered their commercialization [26,28].

## 4. Improving the Properties of PEO−Based Electrolytes

**Figure 2:**
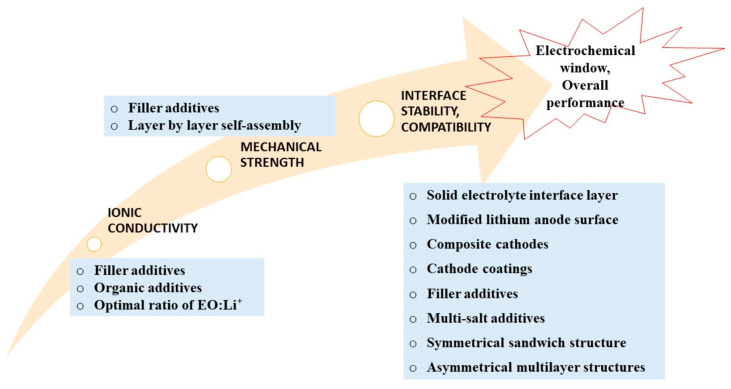
Approaches improving the properties of PEO polymers [30].

Despite the associated drawbacks, PEO is preferable for LIBs compared to poly (acrylonitrile) (PAN), poly (vinylidene fluoride) (PVDF), polycaprolactone (PCL), and polycarbonates [28,29] due to (1) strong electron−donating EO groups, (2) a soft macromolecular backbone, (3) good thermal and mechanical properties, (4) a water−soluble and semi-crystalline state at room temperature [31], as well as lower toxicity [32]. Hence, many advancements and modifications have been made to improve the ionic conductivity, thermal stability, and mechanical stability of these PEO-based electrolytes. Jiang et al. [33] extensively reviewed the synthetic development of PEO-based electrolytes over the past decade using different approaches, such as copolymerization, crosslinking, hyperbranching, blending, composite, ceramic–polymer, salt−soluble polymer, and polymeric single−ion conductors. In Su et al.’s [30] review, they emphasized a few significant approaches to improve the properties of PEO polymers (Figure 2) to desired characteristics.

Additionally, Lee and Kim [34] conferred a few important strategies for improving the properties of PEO−based solid electrolytes, such as the following:

Strategy 1: Inhibit crystallinity via polymer blending, polymer–inorganic composites, block polymers, and hyperbranched polymers.

Strategy 2: Prevent concentration polarization via polyanions in the polymer backbone, organic–inorganic hybrids, and polymers with anion receptor groups.

Strategy 3: Improve mechanical strength via crosslinking and self-healing polymers.

It should be noted that the presence of polar groups such as −O−O, C≡N, or C=O in PEO polymers facilitates the salt dissolution into cations and anions, subsequently speeding up ionic conduction [18]. However, the overall solvation ability can be elucidated by different types of lithium salts (LiX), simultaneously affecting the performance of LIBs. Moreover, inorganic lithium salts (LiPF_6_, lithium tetrafluoroborate (LiBF_4_), etc.) are not suitable to be used owing to their low thermal stability compared to organic lithium salts (lithium bis (trifluoromethanesulfonyl)imide (LiTFSI), lithium bis(fluorosulfonyl)imide (LiFSI), lithium trifluoromethanesulfonate (CF_3_SO_3_Li), etc.). Thus, it is important to identify the optimal type of lithium salts that can alter and significantly increase the performance of PEO−based electrolytes.

Previously, Zhang et al. [35] comprehensively studied the effects of different types of organic lithium salts on the properties of PEO-based electrolytes. It was discovered that lithium salts such as LiTFSI, LiFSI, and CF_3_SO_3_Li possess a high ionic conductivity and are frequently used in LIBs. In their work, they found that the PEO−CF_3_SO_3_Li possessed the lowest ionic conductivity values (0.013 × 10^−4^ S cm^−1^ at 25 ℃; 1.0 × 10^−4^ S cm^−1^ at 60 ℃), which limits its use under large current densities due to the rapidly increasing polarization voltage in the first 30 cycles. PEO−LiTFSI presented the highest discharge capacity, lithium-ion transference number, and ionic conductivities (0.05 × 10^−4^ S cm^−1^ at 25 ℃; 2.4 × 10^−4^ S cm^−1^ at 60 ℃) due to its more flexible structure compared to PEO−LiFSI (0.024 × 10^−4^ S cm^−1^ at 25 ℃; 1.8 × 10^−4^ S cm^−1^ at 60 ℃), which has a more dense and flatter layer [35]. Therefore, this study clarifies the impact of structural characteristics on the transportation of lithium ions, which, in turn, influences ionic conductivity.

Additionally, the salt must have a bulky anion as it has low lattice energy and supports overcoming the coulombic interaction between ions. The mutual dissolution ability of the polymer and salt promotes cation migration via the segmental motion of the polymer chains. Moreover, the polymer chain and salt solvent also influence the dissolution ability of the all-participating species. The solvent must have a high dielectric constant and dipole moment so that the ion association can be hindered. The overall effect of the three key players is the effective number of free ion carriers for transport and the elimination of the ion pair as they do not participate in the conduction. Therefore, to achieve well-balanced properties, a suitable combination of the polymer, salt, and solvent is a vital requirement for the fast ionic conductor [36].

At the core of the effective and sophisticated performance of LIBs, inhibiting crystallinity is the imperative aspect that needs to be improvised in the PEO polymer structure. For instance, Onge et al. [37] proposed the statistical copolymerization method to disrupt the crystallinity of the PEO backbone by introducing a small amount (10 mol%) of non-ethylene oxide comonomer units. In their work, the optimum ionic conductivity of 0.3 × 10^−4^ S cm^−1^ at 25 °C and the Li^+^ transference number of 0.6 were obtained. Hence, their study demonstrated that the presence of propylene oxide (PO) (19%) and 1,2 butylene oxide (BO) (12%) as comonomers results in a reduction in both the size and content of crystalline structures in pure PEO polymer. Moreover, Liu and co−workers [38] worked on a cobalt−based metal–organic framework (Co−MOF) and embedded it within a PEO matrix to improve the segmental motion of PEO. The synergistic dissociation effect created by this filler with LiTFSI and PEO enhanced the dissociation of TFSI^-^ and hopping of Li^+^, respectively. The ionic conductivity of 1.3 × 10^−4^ S cm^−1^ was obtained for PEO/Co-MOF−5% [38]. However, the acid–base pair interaction between metal Co^2+^ and TFSI^-^ ions promotes the separation of LiTFSI molecules. Meanwhile, the nitro group in the organic framework exerts a pulling force on the ether unit of PEO, resulting in the release of Li^+^ ions and enhancing the movement of Li^+^ ions.

Recently, Sun et al. [39] explored the combination of electrospinning and crosslinked techniques on the ionic conductivity of modified PEO electrolytes. In their study, they found that the three−dimensional (3D) nanofiber structure of sodium alginate (SA) and cross-liked calcium ions (Ca^+^) could indeed improve the thermal stability and ionic conductivity (0.69 × 10^−4^ S cm^−1^ at 30 °C) of SA−Ca^+^/PEO electrolytes in a lithium symmetric battery. Their study demonstrates that by adjusting parameters, like spinning dope concentration, voltage, distance, and SA content in the electrospinning process, nanofiber membranes with good morphology and improved electrolyte stability can be produced. Moreover, the crosslinking time significantly impacts the microstructure of nanofibers, leading to increased Ca^2+^ crosslinking and decreased pore size. The addition of Ca^2+^ is also attributed to the formation of metal oxides or carbonates during thermal decomposition. Wang et al. [40] also took advantage of electrospinning techniques by working on electrolytes consisting of lithium alginate (LA), polyacrylamide (PAM), and PEO. The presence of interconnected nanofibers and covalent bonds in the 3D network of LA−PAM−PEO electrolytes inhibited the crystalline structure of PEO, thus improving the Li^+^ conductivity (6.1 × 10^−4^ S cm^−1^), mechanical strength, Li^+^ migration number (0.32), electrochemical window (4.95 V), and low interface resistance (243.8 Ω) compared to pristine PEO and LA−PEO electrolyte, respectively. The study also concluded that the integration of LA and PAM into PEO via the electrospinning process boosted the ionic conductivity by enhancing the dissociation of Li^+^ in the electrolyte. The findings also revealed that the nanofibers exhibited interconnect porous structures that are uniformly distributed, improved mechanical strength, and enhanced adsorption of Li^+^ and TFSI^-^ ions. It is noteworthy that the 3D network topology may offer a convenient transport pathway for Li^+^ in addition to a sizable electrode contact area. These 3D nanofiber membranes also possess smooth surfaces and good thermal stability, which remain steady at high temperatures up to 150 °C [40].

Furthermore, Ritter et al. [41] studied methods for preventing the formation of Li dendrite that could render the ionic conductivity of electrolytes by synthesizing a multielement oxide (MEO) filler, AlTiMgLiO, for CPEs. In their work, the electrochemical window and Li⁺ migration number were found to be 6.18 V and 0.42, respectively. The improvements in terms of electrochemical performance, reducing dendritic growth, and overpotential voltage during galvanostatic cycling proved the potential direction for future studies on using multielement materials as fillers in CPE electrolytes. Moreover, Usta et al. [42] worked on improving the stability window of PEO for a high-potential positive electrode (LiNi_x_Mn_y_Co_z_O_2_) by modifying the reactive terminal group. The replacement of the reactive (−OH) terminal group with (−OCH_3_) and (−C≡N) remarkably increases the ionic conductivity of PEO (4.5 × 10^−4^ S cm^−1^) at room temperature as well as the electrochemical window (>4.18 V). This study adapted the GPEs to synthesize PEO composition at high operating temperatures, taking into account the segmental mobility dependency of Li^+^ conductivity.

Additionally, Li et al. [43] modified the PEO electrolytes with a self-reinforced blend. This method was adhered to by in situ polymerization of ethoxylated trimethylolpropane triacrylate (ETPTA) under UV light in the PEO matrix. The optimum ionic conductivity, electrochemical window, and interface impedance were found to be 1.3 × 10^−4^ S cm^−1^ (55 °C), 5.6 V, and 189 Ω cm^−2^, respectively, for modified PEO (PEO−based blend solid electrolyte (BSPE)). Moreover, this study also revealed the impact of ETPTA loadings on the morphologies associated with the ionic conduction pathway. At high loading of ETPTA, the surface morphologies of PEO−BSPE become rougher and more uneven due to the presence of particles. This ultimately leads to the formation of cracks and stratification, which negatively impacts the performance of LIBs. Conclusively, a sophisticated SPE should possess higher intrinsic properties in terms of ionic conductivity (10^−3^ to 10^−1^ S cm^−1^) [43], Li^+^ transference numbers, electrochemical windows, and mechanical properties (2% offset yield) [44]. Thus, it can be concluded that the combined specialty of higher Li−ion conductivity and high mechanical stability possessed by ISEs and SPEs, respectively, can be founded on CPEs in the presence of optimal types of lithium salts.

## 5. Ion Transport Mechanism in PEO-Based CPEs

In LIBs, the SSEs will act as both electrolytes and separators, allowing the transportation of Li^+^ ions and hindering the physical contact between the electrodes. Thus, to maintain and improve LIB performance, the ideal CPEs should possess the following intrinsic properties [44,45]:

- High ionic conductivity (≥10^−4^ S cm^−1^ at 25 ℃) → facilitates Li^+^ ion transportation.
- Low electronic conductivity (≤10^−12^ S cm^−1^) → avoids the self-discharge of batteries.
- High Li^+^ transference numbers → effectively contribute to Li^+^ diffusion, eliminating concentration gradients, suppressing parasitic reactions, and improving battery performance.
- Wide electrochemical window (0−4.5 V vs. Li/Li^+^) → compatible with the anode and high-voltage cathode.
- Non-flammable, high thermal stability (<5% after 60 min at 90 ℃) → avoids thermal runaway.
- High mechanical strength (2% offset yield) → withstands stress, avoids short-circuiting, and suppresses dendrite growth.
- Small pore size (<1 µm) and moderate porosity (40–60%).
- Thin form (20–25 µm) → reduces the internal resistance, improves energy utilization, improves power densities, and reduces costs.
- Good processability for larger scales.

Generally, CPEs consist of polymers, inorganic fillers, and lithium salts. They not only meet the SPE specialties of processability and flexibility but also bridge the gap between SPEs and ISEs through the incorporation of fillers. Reasonably, the inorganic fillers could act as crosslinking centers to inhibit the polymer crystallization as well as the formation of Lewis acid–base interactions among the surface chemical groups of fillers, lithium salts, and polymers, thus facilitating the dissociation of lithium salts, which eventually increase the ionic conductivity [46]. Commercially, there are two main categories of inorganic fillers: inert and active fillers. However, the functionality of these kinds of fillers depends on the Li^+^ ion conductivity.

The inert fillers can be divided into oxide ceramic fillers (silicon dioxide (SiO_2_), Al_2_O_3_, titanium dioxide (TiO_2_), zirconium dioxide (ZrO)), clay-based materials (montmorillonite, bentonite (Al_2_H_2_O_12_Si_4_)), non-conductive carbon materials (carbon black), minerals (halloysite (Al_2_Si_2_O_5_(OH)_4_), metal–organic frameworks (MOFs), and covalent organic frameworks (COFs). Compared with active fillers, these inert fillers do not have Li-ion attributes and are not directly involved with the Li-ion conduction process [47,48,49]. However, they can help in turning the crystalline structure of a pristine polymer into an amorphous chain segmental by reducing the value of T_g_ of the pristine polymer. Mostly, these types of fillers contribute only to the mechanical strength, thermal stability, and processability of the electrolyte without significantly affecting its electrochemical properties. Moreover, other than reorganizing the polymer backbone structure, these fillers can also indirectly interact with ion pairs, promoting Li^+^ ion dissociation [12]. Whereas the active fillers usually take part in delivering the ionic conductivity and electrochemical reactions, they subsequently have a significant impact on the performance of the electrolyte. These active fillers can be divided into a few types, such as garnet−type, NASICON (Na−type), LISICON (Lithium−type), perovskite-type, lithium nitride (Li_3_N) −type, boron phosphate (BPO_4_) −type, and sulfide−type [31].

**Figure 3:**
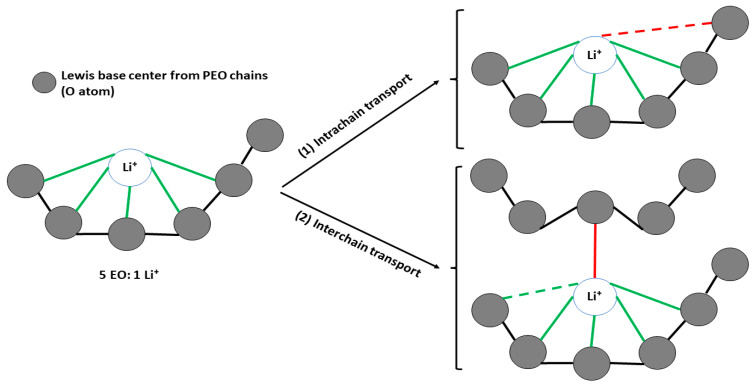
Transport mechanisms of Li^+^ ions in the PEO−LiX matrix (red arrow indicates the type of transport; green arrow indicates the transportation pathway) [31].

Nonetheless, for further understanding, the transport mechanisms of Li^+^ ions on PEO−LiX need to be clearly elucidated. There are two main transport mechanisms involved in PEO−LiX, which are (1) intrachain transportation, involving the five EO−groups in PEO with one Li^+^ ion, and (2) interchain transportation, involving two or more chains of PEO with one Li^+^ ion (Figure 3) [31].

**Figure 4:**
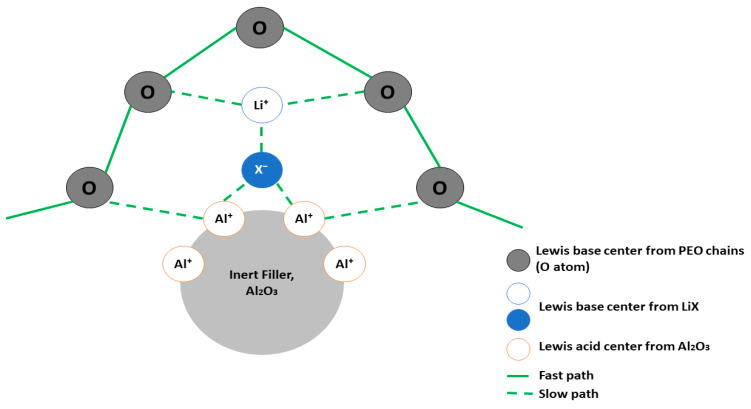
Li^+^ ion transportation in PEO–inert filler composites via the interface of the PEO–inert filler interaction [31].

Specifically, in PEO–inert filler composites, the Li^+^ ion transportation can be divided into (1) the segmental motion of PEO molecular chains via the PEO body (EO groups) and (2) the interface of the PEO–inert filler [31]. The latter transportation pathway has greatly impacted the value of the ionic conductivity of PEO–inert filler CPEs. The PEO–inert filler interface could permanently maintain the amorphous structure, inhibiting the recrystallization of PEO at room temperature, which subsequently increases the ionic conductivity of pristine SPEs. A complex interaction between the Lewis acid center on the surface of inert fillers, Lewis’s base center (O atoms) on PEO chains, and Lewis’s base center (anions) of LiX can assist in releasing freer Li^+^ ions and create the express channels at PEO–inert filler interfaces (Figure 4).

Compared to inert fillers, Li^+^ transportation in PEO–active filler composites can occur through three different pathways: (1) the segmental motion of PEO molecular chains via the PEO body (EO groups), (2) the interface of the PEO–inert filler, and (3) the active filler matrix [31]. As a high concentration of active fillers could provide a continuous pathway for Li^+^ ion transportation, the polymer only acts as a flexible matrix and is not involved in Li^+^ ion diffusion [50], in which the lithium salt can be negligible for the system. For instance, Tao et al. [51] used Li_1.5_A_l0.5_Ge_1.5_(PO_4_)_3_) (LAGP) as an active filler in the PEO matrix with different ratios of PEO:LAGP. The optimal ionic conductivity of 0.3 × 10^−4^ S cm^−1^ was found at a CPE ratio of 6:4 with an electrochemical stability window of >5 V. However, the LAGP not only introduces the Li^+^ conducting site but also improves the amorphous phase of PEO compared to SiO_2_ or Al_2_O_3_. Additionally, three different phases were observed in their study, namely (1) the pure crystalline LAGP phase, (2) the amorphous complexion PEO (LiTFSI) phase, and (3) the transition phase of lithium salt particles and amorphous PEO (LiTFSI). There are a few theories explaining the Li^+^ transportation in active fillers, which are as follows: (1) lower activation energy due to lots of continuous defects in the structures, (2) intensive multiple hopping of ions, and (3) disordered sublattice of active fillers. Moreover, the active fillers can promote the Li^+^ diffusions via two different routes, either vacant or defective defects, also known as Schottky and Flenker defects, respectively [50].

- Schottky defect: an equal number of cations and anions are missing from their normal sites, resulting in an equal number of cation and anion vacancies.
- Frenkel defect: the same ions (cations) leave their normal lattice sites and occupy the interstitial sites.

Previously, Van Gog and Van Huis [52] comprehensively studied the Schottky and Frenkel defects in the structural and electronic properties of magnesium oxide (MgO) {100} ceramic materials. In their work, the surface and bulk properties of Frenkel and Schottky defects were analyzed, and they concluded that both defects are more likely to happen at the surface based on the calculated value of defect formation energies. Moreover, they concluded that the MgO {100} surface could provide a greater dissociation pathway for ions due to the huge band gap and its several electrical defect states. Additionally, it is worth noting that ceramic fillers can act as both inert and active fillers, depending on the specific requirements of the application, the feasibility of conducting Li^+^ ions, or the desired performance in LIBs [50]. Table 3 summarizes the properties of PEO−based CPEs and their corresponding lithium salts.

In Table 3, it can be clearly observed that LiTFSI is most frequently used in studies owing to its well−known high ionic conductivity and solubility in terms of (1) strong electron-withdrawing groups (SO_2_CF_3_), (2) flexible groups (−SO_2_−N−SO_2_−), and (3) high thermal, chemical, and electrochemical stability [30]. From the obtained data, Al_2_O_3_ possessed the highest ionic conductivity of 9.6 × 10^−4^ S cm^−1^ at low temperature (25 °C) compared to other types of inert fillers. However, the specialty possessed by Al_2_O_3_ is paramount to its ability to be an effective inert filler. The Lewis acidic group of Al_2_O_3_ could interact with lithium salt ions (anions), thus promoting the dissolution of anions. Moreover, the basic group of Al_2_O_3_ interacts with Li^+^ ions, hence decreasing the t_+_ value [50]. Conclusively, besides the weakening effect on polymer chains, the simultaneous interaction of inert fillers with Li^+^ ions and anions could enhance the ionic conductivity of pristine PEO.

Alternatively, Li et al. [97] conducted a study involving polypropylene oxide (PPO) with different structures of the LLZO and Al_2_O_3_ inorganic fillers, respectively. From their study, they found that the fibrous fillers offered a longer−distance Li^+^ ion transportation pathway than that formed by the particles. Nonetheless, the fibrous active filler of LLZO showed higher ionic conductivity compared to the fibrous inert filler (Al_2_O_3_), with 4.6 × 10^−4^ S cm^−1^ and 3.4 × 10^−4^ S cm^−1^, respectively. Basically, the operating temperature for commercial LIBs is usually in the range of −20 °C to 60 °C, and outside this range, the battery is susceptible to degradation due to lithium plating and thermal runaway [98]. Yang et al. [44] comprehensively reviewed the effect of fillers in terms of interface, distribution, and interaction in LIBs with respect to their performance properties. It should be noted that the advisable filler content is lower than 50%, so they can be homogeneously distributed within the polymer matrix without blocking the pathways for ionic movement within the electrolyte, leading to increased internal resistance and reduced battery performance.

Liu et al. [99] proposed flexible anion−immobilized modified CPEs to hinder the agglomeration of the fillers. In their study, a silane coupling agent with C=C bonds was adhered to modify the surface of Ta−doped garnet Li_6.4_La_3_Zr_1.4_Ta_0.6_O_12_ (LLZTO) and subsequently grafted with lithium single−ion polymer (lithium (4−styrenesulfonyl) (trifluoromethanesulfonyl) imide, LiSTFSI) to form composite particles of Li@LLZTO. Later, the formed Li@LLZTO was then distributed homogeneously within the PEO matrix to form a CPE of PL@LCSE. High ionic conductivity (15.0 × 10^−4^ S cm^−1^), a wide electrochemical window (~5.3 V), and a high t_+_ (0.77) were obtained in their work. The even distribution of lithium ions in the single−ion polymer layer on the garnet particle surface indeed decreases the crystallinity of the PEO. Furthermore, the formation of lithium dendrites is depressed by the uniform dispersion of Li^+^ ions [99].

Moreover, since the high surface energy of inorganic fillers was believed to disrupt the interfacial contact with the polymer chains, Wang et al. [46] successfully developed a surface positive−charged modification strategy to enhance the interfacial contact of PEO with SiO_2_ nanoparticles. In their work, 0.6 × 10^−4^ S cm^−1^ of ionic conductivity was obtained at an operating temperature of 30 ℃. Interestingly, this modification method not only introduces a greater amorphous structure into the PEO matrix but also enhances the dissociation of lithium salts, activating freer Li^+^ ions to expedite Li^+^ transportation within the PEO matrix [46]. Potentially, the active fillers could deliver a higher ionic conductivity at low operating conditions (25 ℃) than the inert fillers.

## 6. Morphology of Active Fillers

Basically, in solution casting techniques, the inorganic fillers are dispersed into the solution containing the polymer and solvent, and then left for at least 24 h to complete dispersion and become homogeneous. Later, the solution is cast on a glass plate and placed inside the vacuum oven to remove the solvent, leaving a dense structure of the CPEs. This technique offers a simple, cost−effective, and suitable solution for different types of polymer electrolytes. On top of that, the morphology of active fillers plays a crucial role in delivering high−performance CPEs. Each type’s morphology and dimensionality directly correlate with its impact on the battery’s overall performance and stability. The dispersion types of fillers can be classified based on their dimensional structures, such as zero dimension (0D), one dimension (1D), two dimension (2D), or 3D.

**Figure 5:**
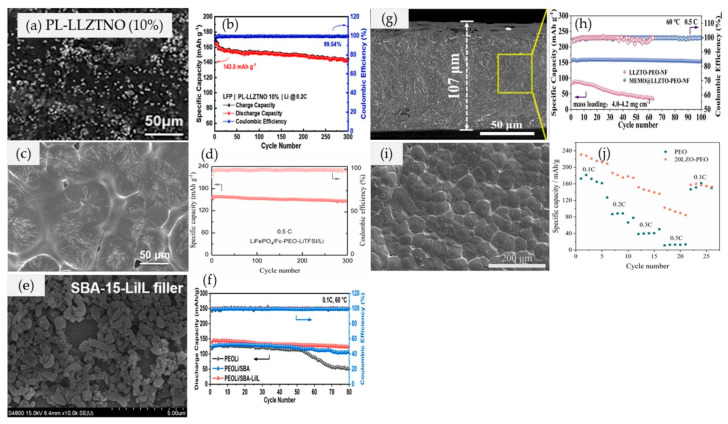
SEM images of active fillers and their respective performance data of (**a**,**b**) PL–LLZTNO (10%) [100], Copyright © 2023 Elsevier B.V., all rights reserved; (**c**,**d**) Fc–PEO [101], Copyright © 2023 Elsevier B.V., all rights reserved; (**e**,**f**) SBA–15LiIL [102], Copyright © 2023 Elsevier B.V., all rights reserved; (**g**,**h**) MEMO@LLZTO–PEO–NF [103], Copyright © 2024 Elsevier B.V., all rights reserved; and (**i**,**j**) LZO–PEO [104], Copyright © 2023 Elsevier B.V., all rights reserved.

For instance, Song et al. [100] used different concentrations of Li_6.5_La_3_Zr_1.5_Ta_0.1_Nb_0.4_O_12_ (LLZTNO (0%, 5%, 10%, and 20%)) in a PEO matrix to construct a CPE (Figure 5a). In their work, they used a powder form (0D) of LLZTNO particles that were directly blended with PEO polymer. The thickness of each electrolyte was found to be 100 ± 5 µm. The optimum concentration of LLZTNO (10%) presented the highest ionic conductivity (0.36 × 10^−4^ S cm^−1^) and a broad voltage window of 4.7 V at room temperature. This electrolyte could retain 143.5 mAh g^−1^ specific discharge capacity and 99.54% coulombic efficiency after 300 cycles (Figure 5b) for LiFePO_4_/PL−LLZTNO (10%)/Li cells. However, the distribution of organic fillers in the PEO matrix is a crucial aspect of Li^+^ ion conduction. A smooth surface in Figure 5a indicates the possibility of the electrolyte making close contact with the electrode, which subsequently decreases resistance and enhances ionic conductivity. Moreover, an optimum loading of filler increases the mechanical strength, which is plausible with extra Li^+^ ion transport pathways [100].

Similarly, Han et al. [101] used ferrocene (Fc) as a novel redox mediator filler in the PEO matrix to optimize the performance of CPEs. They reported that Fc could accelerate the electron transfer and reaction kinetics of the LiFePO_4_ cathode. Additionally, the coordination interactions of Fe−O and Li−cyclopentadienyl ligand cation−π help regulate the Li^+^ ion flux and hinder the growth of lithium dendrites, which subsequently increase the ionic conductivity of the system. The thickness of Fc-PEO CPE was measured to be ~120 µm with a 146.1 mAh g^−1^ discharge capacity and 92.5% capacity retention after 300 cycles (Figure 5c,d) for a Li/Fc−PEO/Li battery. Shen et al. [102], on the other hand, altered the silica clay structure by the hydrothermal method. The final product was found to have a nanopore structure (Figure 5e). The good homogeneity of the mesoporous silica–PEO matrix results in a higher ionic conductivity of 4.3 × 10^−4^ S cm^−1^ (60 ℃) and a specific capacity of 88.4% after 80 cycles (Figure 5f) for Li/LiFePO_4_ cells. The obtained results indicated a reduction in the crystallization area within the PEOLi/SBA−LiIL electrolyte. Li^+^ ion conduction in the PEO polymer primarily occurs in the amorphous phase, facilitated by thermal mobility [58]. Thus, a lower degree of crystallization induced by fillers is beneficial for improving ionic conductivity.

Duan et al. [103] used Li_6.4_La_3_Zr_1.4_Ta_0.6_O_12_ (LLZTO) coated with a 3−methacryloxypropyltrimethoxysilane (MEMO) Janus layer in the PEO matrix. In their work, the ionic conductivity was found to be increased to 2.16 × 10^−4^ S cm^−1^ (30 °C) and the Li^+^ ions transference number was 0.53. This phenomenon is believed to result from the immobilization of lithium salt anions through hydrogen bonding and the interaction of the F-O bond. Nonetheless, they also further investigated the feasibility of MEMO@LLZTO as a function filler in PEO supported by a non-woven fabric (NF) composite electrolyte (Figure 5g). In their work, they found that this electrolyte possessed greater mechanical and thermal stability than the direct blending method while achieving a great cyclability over 4000 h (Figure 5h) in Li/MEMO@LLZTO−PEO−NF/LiFePO_4_ cells. Yang et al. [104] used different loadings of Li_2_ZrO_3_ powder (0D) in the PEO matrix. In their work, they found that the 20 wt.% of Li_2_ZrO_3_ for Li/CPEs/LiNi_0.8_Co_0.1_Mn_0.1_O_2_ (NCM811) battery (Figure 5i) donated the highest values of ionic conductivity (0.07 × 10^−4^ S cm^−1^), electrochemical window (4.2 V), and ionic transference number (0.2). Despite the relatively low ionic conductivity compared to ideal CPEs, the introduction of Li_2_ZrO_3_ is reported to reduce the interfacial resistance between PEO and Li metal, thereby enhancing the stability of these CPEs (Figure 5j). It should be noted that the inclusion of Li_2_ZrO_3_ powder restricts the formation of crystals in PEO, enhances the amorphous region, facilitates the separation and bonding of Li^+^/O^2-^ ions with EO, and subsequently increases the transport of Li^+^ ions [104]. Although a high ionic conductivity (2.17 × 10^−4^ S cm^−1^ at 70 ℃) was obtained at a high loading of Li_2_ZrO_3_ (40%), the induced agglomeration eliminated the feasibility of 40% fillers being further used in LIBs [104].

**Figure 6:**
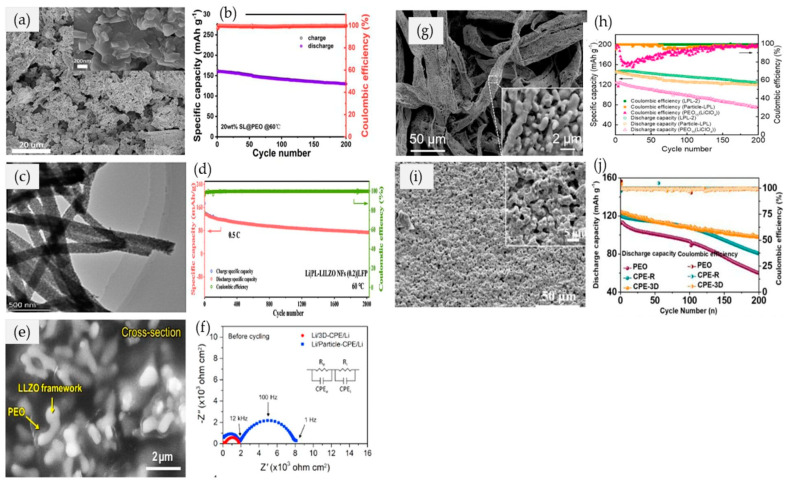
SEM images of active fillers and their respective performance data of (**a**,**b**) SL–LLZAO [105], Copyright © 2022 Elsevier B.V., all rights reserved; (**c**,**d**) 3D LLZO nanofiber [106], Copyright © 2024 Elsevier B.V., all rights reserved; (**e**,**f**) garnet LLZO [107], Copyright © 2018 Elsevier B.V., all rights reserved; (**g**,**h**) 3D LLZAO [108], Copyright © 2019 Elsevier B.V., all rights reserved; and (**i**,**j**) 3D LATP [77], Copyright © 2022 Elsevier B.V., all rights reserved.

Additionally, according to Cheng et al. [105], sheet-like (SL) LLZAO (Li_6.25_La_3_Zr_2_Al_0.25_O_12_) (Figure 6a) was successfully synthesized by the bottom−up method to upgrade the PEO matrix (SL@PEO) for enhanced ionic conductivity of CPE and battery performance. The calculated ionic conductivity of 20 wt.%  SL@PEO possesses the highest ionic conductivity of 0.43 × 10^−4^ S cm^−1^ at room temperature. LiFePO_4_/SL@PEO/Li all−solid−state batteries show a high initial specific capacity of 160 mA h g^−1^ and 81% capacity retention after 200 cycles at 60 °C (Figure 6b). It also has an initial specific capacity of 150 mA h g^−1^ and 80% capacity retention after 210 cycles at 50 °C. Two−dimensional sheets like LLZAO (SL) fabricated via the facile sol–gel method with carbon nanodots (CDs) as initial nucleation sites can provide more barriers for the electrolyte to suppress the lithium dendrites’ growth. The batteries made of all−solid−state LiFePO_4_/SL@PEO/Li demonstrate excellent cycling stability and rate capability. However, the ionic conductivity of CPEs can still be disrupted by the agglomeration of 0D nanoparticles or crossing junctions of 1D nanofibers within the polymer matrix. Thus, a 3D active filler is believed to inhabit agglomeration as well as lithium dendrite formation, in addition to the effective fast conductive pathways for Li^+^ ions via a percolated network [77].

The electrospinning process is one of the methods to create a more flexible structure of inorganic fillers with high surface area and porosity. The optimization of spinning parameters is crucial in determining the desired structure of electrospun 3D non−woven fibers. For instance, Teng et al. [106] electrospun a precursor solution containing polyvinylpyrrolidone (PVP) and garnet-type LLZO with different molar amounts of In^3+^. The electrospinning process was adhered at a voltage of 16 kV, and the distance from the needle tip to the collector was 18 cm. Next, the calcination process was performed to obtain a powder form of LLZO (Figure 6c). In their work, 0.2 molar amounts of In^3+^ in Li_7_La_3_Zr_2_O_12_ (LLZO) nanofibers provided a stable cubic−phase 3D structure, which subsequently increased the lithium vacancy concentration and the Li^+^ ion transportation pathway within the crystal’s pores. The increments in ionic conductivity (9.4 × 10^−4^ S cm^−1^ at 60 ℃), electrochemical window (5.2 V), and transference number (0.19) were induced, respectively. This electrolyte could retain ~100% coulombic efficiency after 2000 cycles in Li/LiFePO_4_ cells (Figure 6d). Nonetheless, other than the processability of electrospinning, the tangle of controlling parameters makes it complicated to sustain many studies.

Thus, different approaches have been persuaded to decipher the associated problems with the aforementioned method by modifying the structure of fillers. Previously, Bae et al. [107] synthesized a 3D interconnected garnet, Li_6.28_La_3_Zr_2_Al_0.24_O_12_ (LLZO), framework via nanostructured hydrogel as a template (Figure 6e). In a polytetrafluoroethylene (PTFE) dish, the PEO−LiTFSI solution completely filled up the pores of the 3D LLZO framework by immersion precipitation. However, continuous lithium−ion conduction was achieved due to the integrated structure of 3D LLZO, which subsequently increased the mechanical and thermal stability of the CPEs. The ionic conductivity was found to be 0.9 × 10^−4^ S cm^−1^ and ~10^−3^ S cm^−1^ at 25 ℃ and 60 ℃, respectively. Next, Fu et al. [108] employed the cleanroom wiper as a 3D template in delivering a 3D Li_6.28_La_3_Zr_2_Al_0.24_O_12_ (LLZAO) framework (Figure 6g). In their work, they found that the 3D LLZAO not only inhibited the growth of lithium dendrite but also simultaneously increased the ionic conductivity (2.2 × 10^−4^ S cm^−1^ at 30 ℃) up to 30.7 folds greater than pristine PEO−LiClO_4_ by the fast ionic transportation pathway. The 3D LLZAO−PEO−LiClO_4_ recorded 143.8 mAh g^−1^ of initial discharge−specific capacity and 86% capacity retention at 60 ℃ after 200 cycles (Figure 6h) in the LiFePO_4_/Li battery system, respectively.

Nonetheless, Wang et al. [77] used a sodium chloride (NaCl) powder as a template in synthesizing a 3D Li_1.3_Al_0.3_Ti_1.7_(PO_4_)_3_ (LATP) porous framework (Figure 6i). The immersion and precipitation processes are repeated to completely fill the porous structure of LATP with the PEO−LiTFSI matrix. Furthermore, this study investigated the effect of different NaCI loadings on the morphology of the 3D LATP framework and concluded their effect on Li^+^ ion transportation behavior. In this study, the loadings of NaCI varied from 50 to 70 wt.%, which contributed to the loosely packed morphologies of 3D LATP frameworks. At 50 wt.% of NaCI, branches with lengths ranging from 3 to 6 µm formed, with pore diameters of 1 to 4 µm. A shorter length of ~3 µm was induced when the NaCI content was increased to 60 wt.%, in addition to a higher pore diameter size (~6 µm). Meanwhile, at 70 wt.% of NaCI, perfect branch length and pore diameter (5–20 µm) were induced, which simultaneously provided a good pore channel for the inclusion of the PEO polymer [77]. Thus, the 3D LATP−PEO with 70 wt.% of NaCl had an ionic conductivity of 7.5 × 10^−4^ S cm^−1^, which was 7 folds higher than the pristine PEO−LiTFSI electrolyte. After 200 cycles, the prepared CPEs possessed a higher discharge capacity of 98 mAh g^−1^ and a retention value of 80% for the LiFePO_4_/Li cell at a current density of 1C (60 ℃) (Figure 6j).

## 7. Characterization Techniques and Performance Evaluation of CPEs

The synthesis of CPEs involves numerous procedures that determine their overall performance, for which a detailed understanding of their features is critical. Furthermore, to meet the difficult criteria of diverse energy storage applications, a thorough examination is required. Characterization techniques like structural analysis, thermal analysis, morphological investigation, and electrochemical analysis are critical in unraveling the complexities of CPEs and providing insights into their composition, structure, and electrochemical behaviors. Thus, this chapter emphasizes the characterization of CPEs.

Moreover, advanced characterization techniques are indispensable tools for understanding and optimizing PEO–active filler CPEs, particularly concerning their impact on lithium-ion battery performance. These techniques bridge the gap between theory and application, providing valuable insights into various aspects such as electrochemical performance evaluation, performance comparison, and prospects [109]. In the realm of electrochemical performance evaluation, advanced characterization techniques offer a detailed assessment of ionic conductivity, which is a significant parameter influencing battery performance. Techniques such as impedance spectroscopy and conductivity measurements enable researchers to quantitatively analyze the influence of composite components, including active fillers like ceramic nanoparticles, on ion transport properties. By delving into the nanoscale interactions within the composite electrolyte, researchers can tailor its composition to enhance ionic conductivity, thereby optimizing battery performance [110].

Performance comparison between PEO–active filler CPEs and traditional polymer electrolytes is facilitated by advanced characterization techniques. Through techniques like X−ray diffraction and scanning electron microscopy, researchers gain insights into the microstructure and composition of the composite electrolyte [111]. This comparative analysis elucidates how the unique morphology and composition of composite electrolytes outperform their traditional counterparts, providing valuable insights into their potential for practical applications in lithium−ion batteries [112]. Moreover, advanced characterization techniques shed light on the effect of filler type and concentration on composite electrolyte performance. Techniques such as transmission electron microscopy (TEM) and X-ray photoelectron spectroscopy (XPS) allow researchers to analyze the nanoscale distribution and chemical composition of fillers within the polymer matrix. This in−depth understanding enables researchers to correlate filler characteristics with electrochemical performance, guiding the optimization of filler type and concentration for improved battery performance [113].

The impact of processing parameters on composite electrolyte performance is also elucidated through advanced characterization techniques. Techniques such as in situ spectroscopy provide real−time insights into structural changes during fabrication processes like solution casting or hot pressing. By understanding how processing parameters influence the microstructure and properties of the composite electrolyte, researchers can optimize fabrication methods to enhance battery performance and reliability [114]. Exploring potential applications and prospects of PEO–active filler CPEs is also facilitated by advanced characterization techniques. Techniques such as electrochemical impedance spectroscopy and differential scanning calorimetry provide crucial data for predicting the behavior of composite electrolytes in real−world applications. This enables researchers to tailor composite electrolytes for specific applications, ranging from portable electronics to electric vehicles and grid energy storage, while also considering technological and commercial implications [20]. Ultimately, advanced characterization techniques serve as indispensable tools for unraveling the complexities of PEO–active filler CPEs and their impact on lithium−ion battery performance. By providing detailed insights into electrochemical performance, filler characteristics, processing parameters, and potential applications, these techniques drive advancements in energy storage technologies and pave the way for future innovations in the field.

### 7.1. Determining the Components in CPEs

The methodical procedure for determining components in CPEs is critical for quality control, research, and guaranteeing compliance with specified material specifications. This entails researching and evaluating how various components inside a composite material contribute to its overall qualities and performance. Composite materials are engineered materials made up of two or more separate components with distinct qualities that are combined to form a material with improved properties. Component analysis in CPEs is often conducted using a combination of advanced analytical techniques. Spectroscopy is a key method, and Fourier-transform infrared spectroscopy (FTIR) is extensively employed to determine the chemical vibrations of CPE components. This method enables the precise identification of functional groups and molecular structures in a substance.

**Figure 7:**
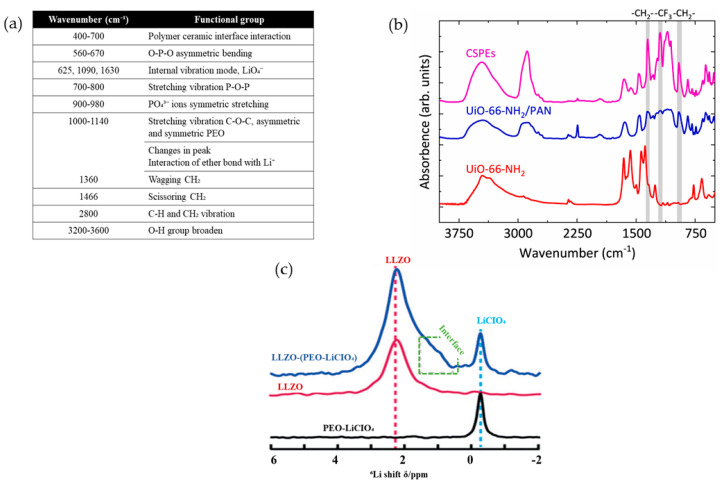
(**a**) FTIR data of PEO, PEO–LiCIO_4_, and LiSn_2_(PO_4_)_3_–(PEO–LiCIO_4_) [115]; (**b**) FTIR spectrum of CSPEs [116], Copyright © 2022 Elsevier B.V., all rights reserved; and (**c**) ^6^Li NMR spectrum of LLZO–(PEO–LiCIO_4_) [117], Copyright © 2016 Elsevier B.V., all rights reserved.

Zhao et al. [115] examined the functional groups of PEO−LiTFSI and zeolitic imidazolate framework−67 (ZIF−67) for lithium-ion battery applications via FTIR analysis. The data (Figure 7a) reported the stretching vibrations of C−H and C−O−C bonds at peaks 2878 cm^−1^ and 1094 cm^−1^ of PEO, respectively. The addition of ZIF−67 slightly shifted the peak to 1095.33 cm^−1^ for C−O−C bonds, which was mainly attributed to the weak interaction of PEO and Li^+^, simultaneously providing freer Li^+^ ions in the composite matrix. Examples of FTIR spectra can be found in Figure 7b, which was adopted from work by Li et al. [116]. From the obtained spectroscopy, the CSPEs have a distinct peak at 1196 cm^−1^, which corresponds to the stretching triplet modes of −CF_3_. The presence of the −CF_3_ group in an electrolyte has a substantial impact on its chemical stability and ionic conductivity. It improves stability and decreases reactivity with lithium metal materials. Spectroscopy provides insights into the surrounding conditions and interconnections of these groups.

Apart from that, the utilization of nuclear magnetic resonance (NMR) spectroscopy is useful in understanding the molecular structure, arrangement, and chemical environment of certain nuclei in a substance. Due to the high sensitivity of NMR spectra, the Li−ion position within the CPEs can be differentiated either in the polymer phase, ceramic phase, or interfacial polymer–ceramic phase [117]. For instance, Figure 7c depicts a high−resolution ^6^Li NMR spectrum of LLZO−(PEO−LiClO_4_). As seen in Figure 7c, the initial peak observed at δ = −0.2 ppm indicates the presence of LiClO_4_ in the PEO−LiClO_4_ polymer matrix. Another peak at δ = 2 ppm represents the Li in pure cubic LLZO fillers, whereas an inclined peak at ~δ = 1.4 ppm corresponds to the Li positioned at the interface of PEO and LLZO. Other researchers, for example, Fu et al. [118] also elucidated some reviews on the ionic transportation mechanism within the composite polymer electrolyte based on IR and NMR spectra, respectively. Lewis’s base center (O atoms) on PEO chains, and Lewis’s base center (anions) of LiX can assist in releasing freer Li^+^ ions and create the express channels at PEO–inert filler interfaces (Figure 7c).

### 7.2. Morphological and Structural Study

**Figure 8:**
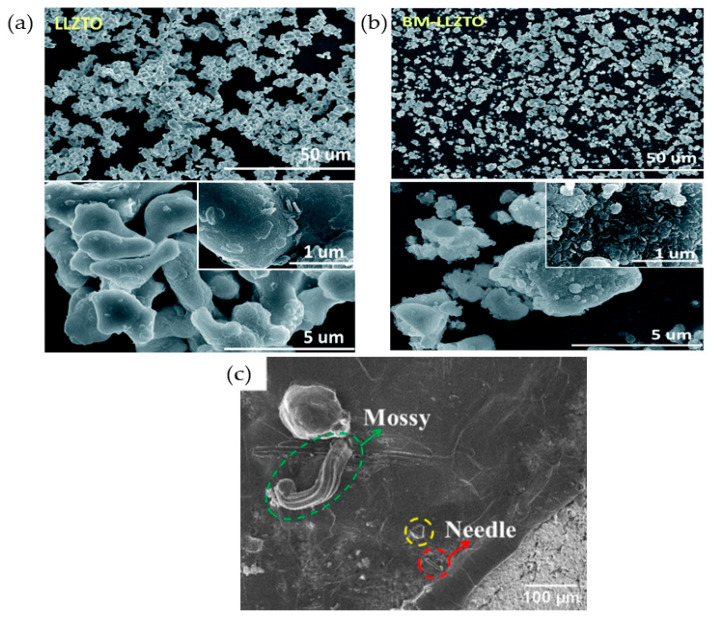
(**a**,**b**) FESEM images of LLZTO [120] and (**c**) SEM image of lithium dendrites [121], Copyright © 2020, Springer Nature, all rights reserved.

Morphological study by field emission scanning electron microscopy (FE−SEM), scanning electron microscopy (SEM), energy dispersive X-ray (EDX/EDS), atomic force microscopy (AFM), and X-ray diffractometer (XRD) are the second major characterizations for CPEs. These characterizations can determine the thickness, pore size, distribution, porosity, and tortuosity of the materials. For instance, He et al. [119] investigated the morphology and structure of composite electrolyte polyethylene oxide and different loadings of Li_6.4_La_3_Zr_1.4_Nb_0.6_O_12_ (LLZNO). From their observation, at higher loadings of the LLZNO fillers, the particles tend to be agglomerated, simultaneously turning the smooth surface into a coarse surface. Nonetheless, a thickness of 100 µm was recorded from FESEM analysis. A further examination was conducted by EDS for elemental analysis of the sample, indicating the homogenous distribution of LLZNO (zirconium, niobium, and lanthanum) within the PEO matrix [119]. Apart from that, Song et al. [120] also applied FESEM and EDX mapping in order to investigate the effect of LLZTO particle size after being treated via the ball−milling (BM) method (Figure 8a,b). Notably, the particle size of BM−LLZTO particles after 30 min of treatment (5.75 mm) is smaller compared to pristine LLZTO particles, yet these BM−LLZTO show agglomeration between primary particles with nanosized particles. Additionally, after 80 min of treatment, the size of BM−LLZTO becomes larger (9.96 mm) than pristine LLZTO particles due to excessive agglomeration. Moreover, Golozar et al. [121] also examined the lithium dendrite by SEM analysis, and they managed to observe two different types of dendrites presented on the ceramic and symmetrical Li−LLZO−Li cells which were “mossy” and “needle” types (Figure 8c).

**Figure 9:**
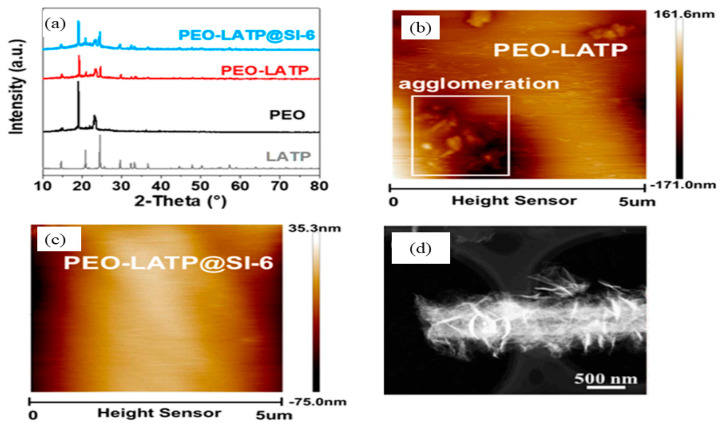
XRD patterns of materials in PEO–LATP@SI–6 (**a**); top AFM view of PEO– LATP (**b**); PEO–LATP@SI–6 (**c**) [122], Copyright © 2023 American Chemical Society, all rights reserved; and HRTEM nanoflake structure of MIL–53 (Al) (**d**) [123], Copyright © 2024 Elsevier B.V., all rights reserved.

Meanwhile, Rong et al. [122] examined the XRD pattern and AFM images of the PEO−LATP@SI−6 composite electrolyte. Figure 9a depicts the XRD patterns of all components in the PEO−LATP@SI−6 composite electrolyte. Both the intensity peaks of PEO−LATP and PEO-LATP@SI-6 are much lower than those of PEO, corresponding to the reduction in crystallinity. Nonetheless, the AFM (Figure 9b,c) showed that the PEO−LATP@SI−6 possessed a flat, smooth surface compared to the agglomeration present on the PEO−LATP surface. Thus, these two characterizations have proven the improvement in ionic conductivity from 0.52 × 10^−4^ S cm^−1^ to 1.15 × 10^−4^ S cm^−1^ of PEO−LATP and PEO−LATP@SI−6 [122], respectively. Nonetheless, the high-resolution TEM (HRTEM) analysis was used by Wang et al. [123] to clearly investigate the structure of MIL−53 (Al) nanofibers (Figure 9d).

### 7.3. Stability of CPEs

**Figure 10:**
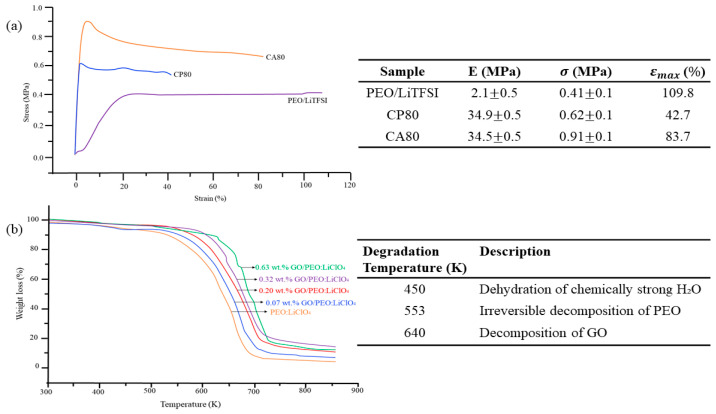
Stability measurement of CPEs: (**a**) stress–strain curve and (**b**) TGA curve with their tabulated data [124,125].

Despite the composition and morphology of CPEs, stability assessment is also crucial since it impacts battery safety, performance, and processability. Basically, mechanical strength and thermal stability are two important parameters for investigating the stability and decomposition of materials. Figure 10a examines the stress–strain curve for composite PEO, consisting of LLZO and LiTFSI salt. Ghorbanzade et al. [124] made some amendments to the properties of LLZO via the heat treatment method, and the samples were disseminated as CP80 and CA80, representing pristine and heat-treated LLZO, respectively. In Figure 10a, the mechanical strength is improved as the LLZO is added, which proves that the ceramic or inorganic material acts as mechanical reinforcement. Next, the thermal stability measurement was conducted by Khan et al. [125] on PEO/LiClO_4_ in the presence of graphene oxide (GO). In Figure 10b, the first degradation of PEO (450 K) increased as the loading of GO increased. However, a percentage of 0.63 showed an optimum degradation of GO at 640 K. Further descriptions can be found in the table.

### 7.4. Electrochemical Properties

**Figure 11:**
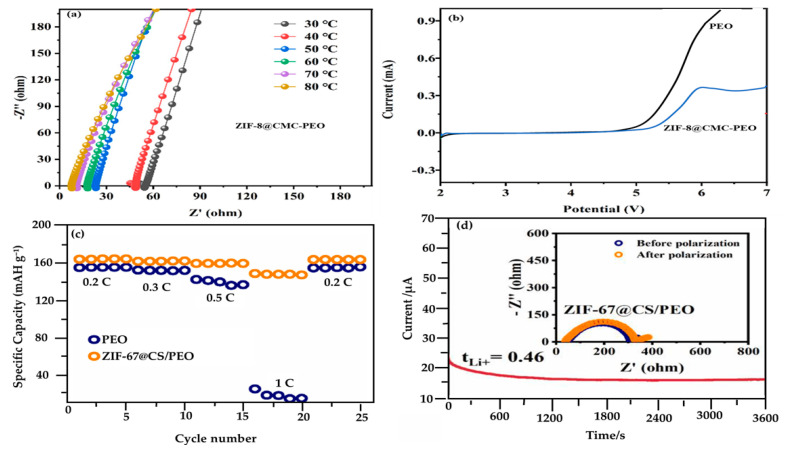
(**a**) Ionic conductivity; (**b**) electrochemical window of PEO and ZIF–8@CMC–PEO, respectively [126], Copyright © 2023 Elsevier B.V., all rights reserved; and (**c**) transference number and (**d**) charge–discharged performance of PEO and ZIF–67@CS/PEO, respectively [127], Copyright © 2024 Elsevier B.V., all rights reserved.

In LIBs, characterization of the electrochemical properties is crucial because it can determine the usability of LIBs for further commercialization. The fundamental exploration of various properties can be elucidated by investigating lithium ionic conductivity, electrochemical window measurement, transference number, interfacial stability, cycling, and charge–discharge performance. Noteworthy, exemplary work by Wei et al. [126] summarizes two of the important electrochemical tests for lithium-ion batteries as depicted in Figure 11a,b. In their work, they found that the optimum loading of ZIF−8@CMC within the PEO matrix was 20% (6.37 × 10^−4^ S/cm^2^ at 60 ℃) (Figure 11a). Above this point, the filler did not have any significant effect on the proton conductivity when the applied temperature was higher than the melting point of the PEO polymer.

It is suggested that above the melting point of PEO, the intrinsic properties of the molten polymer dominate the ion transport process, rendering the contribution of fillers to proton conductivity less significant. The increased chain mobility, uniform filler dispersion, and the inherent conductive mechanisms of the amorphous polymer phase diminish the relative impact of fillers on the overall conductivity.

According to the Arrhenius equation, the activation energy of composite ZIF–8@CMC–PEO (0.207 eV) at 60 ℃ was lower than that of pristine PEO (0.282 eV), thereby a lower energy barrier is necessary for Li−ion transportation in the composite electrolyte. Apart from that, according to Stephen and Nahm [128], it was also important to study the electrode materials since their good interfacial compatibility with the electrolyte can determine the number of Li-ions transported. The linear sweep voltammetry (LSV) test was held to measure the composite electrochemical window. This testing was important in determining the electrochemical stability of the materials for LIBs. Figure 11b indicates that the ZIF−8@CMC−PEO possessed a higher potential of ~5 V compared to pristine PEO (~4.3 V), thereby fulfilling the requirement for a LIB electrolyte with a higher electrochemical window of >4.5 V [126]. Meanwhile, the measurement of the transference number for Li−ions was conducted using the method of timing current at a polarization voltage of 10 mV and an 8000 s polarization time for the ZIF−8@CMC−PEO electrolyte [126].

Meanwhile, the transference number (t_Li_^+^) and charge–discharged performance were collected from the study by Song et al. [127] on the ZIF−67@CS/PEO electrolyte. In Figure 11c, the calculated Li-ion transference number of composite electrolytes was 3.8 folds higher than pristine PEO (0.12). However, the improvement in t_Li_^+^ can be attributed to the persistence and accessibility of CO^2+^ metal sites on ZIF−67@CS. These sites have the ability to form complexes with anions (TFSI^−^) that are present in the electrolyte pore channels. These interactions have accelerated the dissociation and movement of LI^+^ ions, resulting in the formation of a rapid and uninterrupted pathway for Li^+^ conduction [127]. Moreover, Figure 11d demonstrates the charge–discharge performance at different rates of cycle (0.2, 0.3, 0.5, and 1C, respectively). Figure 11d clearly shows that the high discharging capacities obtained at ~164.8 mAh g^−1^ at 0.2C rates at that composite electrolyte could withstand a long-cycle life of 2500 h at 30 ℃ and could prevent lithium dendrite development [127]. Foremost, the electrochemical property characterizations are the most crucial in LIBs since they can recognize the upfront problems such as electrode degradation, capacity, and voltage fade in order to increase battery longevity. Table 4 summarizes all the characterization techniques and their functionalities. Conclusively, the investigation of electrochemical properties contributes to the advancement of novel materials and electrode configurations, resulting in the development of battery technologies that are more sustainable and safer.

## 8. Factor Influencing Performance

PEO–active filler CPEs wield a profound influence on lithium−ion battery performance, impacting critical aspects such as cycling stability, rate capability, capacity retention, and longevity. The integration of active fillers, particularly ceramic nanoparticles, enhances the cycling stability of batteries. This is attributed to the conductive network formed by these fillers, effectively mitigating issues like electrode–electrolyte detachment during dynamic charge–discharge cycles. The improved cycling stability is pivotal for extending the lifespan of lithium−ion batteries, ensuring sustained and reliable performance over numerous cycles [129]. Moving beyond cycling stability, PEO–active filler CPEs showcase notable advantages in rate capability and power density. The conductive pathways established by active fillers facilitate rapid ion transport, enabling the battery to deliver high power outputs, especially under demanding conditions. This characteristic is particularly valuable in applications where quick charging and discharging are essential, contributing to the adaptability of these electrolytes across diverse scenarios [130].

The delicate balance achieved through the optimal type and concentration of active fillers plays a crucial role in enhancing capacity retention and the overall longevity of lithium-ion batteries. The conductive network within the composite electrolyte maintains efficient ion transport, preventing degradation mechanisms and supporting prolonged battery life. This feature is significant for applications requiring reliable and long−lasting energy storage solutions, ensuring that the batteries maintain their performance characteristics over extended periods [131]. In a comparative analysis with conventional electrolytes, PEO–active filler CPEs demonstrate notable advantages. Enhanced ionic conductivity and improved thermal stability contribute to superior battery performance. Safety considerations, such as a reduced risk of thermal runaway, position these composite electrolytes as promising alternatives. Moreover, performance metrics, including capacity, energy density, and cycle life, often favor composite electrolytes [132]. The conductive network formed by active fillers addresses limitations seen in conventional electrolytes, positioning PEO–active filler composite polymer electrolytes as contenders for applications where superior performance is paramount [133].

Practical applications benefit significantly from the versatility of PEO–active filler CPEs. Their improved safety profile, along with favorable performance metrics, makes them suitable for various applications, including portable electronics, electric vehicles, and grid energy storage. The adaptability of these composite electrolytes positions them as promising solutions, capable of addressing the evolving needs of diverse energy storage systems. In conclusion, the multifaceted impact of PEO–active filler CPEs on lithium-ion battery performance underscores their potential to shape the future landscape of energy storage technologies [134].

## 9. Conclusions and Future Outlooks

This comprehensive analysis has elucidated the remarkable advancements and promising prospects in PEO–active filler CPEs for LIBs. The fusion of active fillers into the PEO framework has demonstrated a substantial augmentation in the ionic conductivity and electrochemical efficacy of composite polymer electrolytes, surmounting the constraints linked to pure PEO electrolytes. A diverse array of active fillers has been meticulously explored for their role in enhancing the transport properties of lithium ions within the electrolyte framework. The exploration of the mechanisms that govern the improvement in ionic conductivity and lithium−ion transportation in composite polymer electrolytes based on PEO offers valuable insights for the deliberate development and enhancement of electrolyte materials for lithium−ion batteries. The incorporation of active fillers in PEO−based composite electrolytes can significantly improve their ionic conductivity. This is crucial for faster charging and discharging rates in lithium−ion batteries. Active fillers such as ceramic nanoparticles or nanofibers can enhance the mechanical strength and stability of the composite electrolyte, leading to better performance and longer cycle life of the batteries. Moreover, composite electrolytes with active fillers can exhibit a wider operating temperature range compared to pure PEO electrolytes. This is important for applications in varying environmental conditions. Despite notable advancements, there are still several obstacles to overcome in order to fully unleash the potential of PEO–active filler composite polymer electrolytes in real-world battery applications, including interface compatibility, mechanical stability, long-term durability, dendrite formation, and flexibility.

Anticipating future endeavors, it is imperative that additional exploration be directed toward the creation of innovative active agents, refined synthesis approaches, and meticulous characterization methodologies in order to customize the attributes of composite polymer electrolytes to meet distinct battery prerequisites. Furthermore, the incorporation of emerging technologies, such as solid−state electrolytes and additive manufacturing techniques, holds the potential to facilitate the achievement of superior efficiency and safety in LIBs, accompanied by heightened energy capacity and longevity. Future advancements may incorporate active fillers that target dendrite suppression, such as dendrite−blocking polymers or nanostructured materials designed to inhibit dendrite growth. Moreover, exploring the nanostructures of active fillers can lead to battery performance due to higher surface area, improved dispersion, and enhanced ionic conductivity within the polymer matrix. There is a growing need for flexible and stretchable electronics, which requires the development of PEO−based composite electrolytes that can maintain high ionic conductivity even when under mechanical deformation. Active fillers that possess flexible or elastic properties could be instrumental in achieving this objective. Future advancements in composite electrolytes will prioritize sustainability and environmental impact by developing bio−based or recyclable fullers and eco−friendly manufacturing processes. In essence, this critique highlights the significance of ongoing exploration and creativity in the realm of PEO–active filler composite polymer electrolytes for lithium−ion batteries, offering a blueprint for forthcoming progress in the advancement of cutting−edge energy storage technologies.

## Figures and Tables

**Table 1:** The development of ISEs and SPEs in the past few centuries [20].

Year	ISEs	SPEs
1800s	First introduction of PbF_2_, Ag_2_S, yttrium oxide (Y_2_O_3_) −doped zirconium dioxide (ZrO_2_), silver iodide (Agl)	NA
1960s	Introduction of beta−aluminum oxide (β−Al_2_O_3_) −based ISEs Introduction of sodium sulfide (Na−S) batteries with Na^+^−β−Al_2_O_3_	NA
1970s	Discovery of Na superionic conductor (NASICON) and lithium superionic conductor (LISICON) −based ISEs Introduction of hydride−type ISEs	Discovery of PEO SPE and NASICON - Homopolymer
1980s	NA	Introduction of sodium nickel chloride (Na−NiCl_2_) batteries with molten sodium tetrachloroaluminate (NaAlCl_4_)/Na^+^−β−Al_2_O_3_ SSE - Polymer with plasticizer - Composite polymer - Single−ion conductor - Copolymers and crosslinked polymers
1990s	Non−crystalline lithium oxonitridophosphate (LiPON), sulfides, (anti) perovskite, and garnet for ISEs	The first thin−film battery with the development of LiPON
2000s	NA	Revival of interest in Li metal
2010s	NA	Development of Bolloré Bluecar using a Li−polymer battery

NA: not available.

**Table 2:** Properties of PEO at different Mw values [31].

Low M_w_ PEO (Ideal)	Increment of M_w_	High M_w_ PEO
Low T_g_	Increase T_g_	High T_g_
High amorphous proportion	Reduce the amorphous region	Semi-crystalline
Good ionic conductivity	Reduce ionic conductivity	Limited ionic conductivity at room temperature
Low thermal stability	Increase viscosity	-
Low mechanical strength	Increase mechanical strength	Regularity of PEO chains destroyed by the following: (a) Grafting to construct a comb-like structure. (b) Inserting to make block structure and crosslinking.

**Table 3:** Studies on PEO–inorganic CPEs with their properties.

Filler Type	Lithium Salt	Ionic Conductivity (×10^−4^ S cm^−1^)	^1^ EW (V)	Transference Number (t_+_)	Discharge Capacity (mAh g^−1^)	Ref.
*Inert Filler: Ceramic Oxide-Type*						
Aluminum oxide (Al_2_O_3_)	LiTFSI	9.60 (25 °C)	5.00	0.81	640 (0.1 C)	[53]
Ti^3+^-doped TiO_2_	LiTFSI	1.00 (30 °C)	5.50	0.36	151 (60 °C, 0.1 C)	[54]
*Inert Filler: Ferroelectric-Type*						
Barium titanate (BaTiO_3_)	LiTFSI ^2^ LiClO_4_	1.30 (30 °C) 12.0 (70 °C)	4.00 -	- 0.37	-	[55] [56]
Lithium niobate (LiNbO_3_)	LiCF_3_SO_3_	2.00 (85 °C)	-	0.52	-	[56]
*Inert Filler: Porous-Type*						
Aluminum benzene tricarboxylate (Al-BTC)	LiTFSI	10.0 (30 °C)	>3.8	0.55	-	[57]
Zirconium benzene dicarboxylate (UiO-66)	LiTFSI LiTFSI	13.0 (30 °C) 29.0 (60 °C)	4.50 4.30	0.35 0.52	~151 (60 °C, 0.5 C) -	[58] [59]
Vinyl-functionalized MOF nanoparticles (UIO-66-NH_2_)	LiTFSI	63.0 (60 °C)	4.97	0.72	141.2 (0.1 C)	[60]
Aluminum terephthalate (Al-TPA)	LiTFSI	1.00 (60 °C)	>3.0	-	130 (0.1 C)	[61]
Aluminosilicate zeolite (SSZ-13)	LiTFSI	170.0 (60 °C)	4.65	0.84	156.63	[62]
MIL-53 (Al)	LiFSI	34.0 (120 °C)	5.10	0.34	103.5 (120 °C, 10 C)	[63]
*Inert Filler: Mineral-Type*						
Kaolinite (Al_2_Si_2_O_5_(OH)_4_)	LiTFSI	1.10 (25 °C)	6.35	0.4	919 (0.3 C)	[64]
Suanite (Mg_2_B_2_O_5_)	LiTFSI	1.50 (40 °C)	4.75	0.44	150 (50 °C, 0.2 C)	[65]
*Active Filler: Garnet-Type*						
Li_6.25_Al_0.25_La_3_Zr_2_O_12_ (LLZO) Li_7_La_3_Zr_2_O_12_ (LLZO)	^2^ LiClO_4_ LiTFSI	3.00 (24 °C) 2.40 (25 °C)	5.00 6.00	- -	122 (70 °C, 0.1 C) 158.8 (60 °C, 0.5 C)	[66] [67]
Li_7_La_3_Zr_2_O_12_ (LLZO)	LiTFSI ^2^LiCIO_4_	0.90 (25 °C) 4.40 (50 °C)	5.50 6.00	- -	170 (1 C) 166 (55 °C, 0.02 C)	[68] [69]
Li_6.4_La_3_Zr_2_Al_0.2_O_12_ (LLZO)	LiTFSI	2.50 (30 °C)	6.00	-	-	[70]
Li_6.55_Ga_0.15_La_3_Zr_2_O_12_ (LLZO)	LiTFSI	4.50 (70 °C)	-	-	-	[71]
Li_6.75_La_3_Zr_1.75_Ta_0.25_O_12_ (LLZTO)	^2^ LiClO_4_ LiTFSI	5.00 (25 °C) 0.11 (25 °C)	- 5.50	- -	120 (0.3 C) 155 (60 °C, 0.1 C)	[72] [73]
Li_6.4_La_3_Zr_1.4_Ta_0.6_O_12_ (LLZTO)	LiTFSI	2.10 (30 °C)	4.75	-	153.3 (60 °C, 0.05 C)	[74]
*Active Filler: NASICON-Type*						
Li_1+x_Al_x_Ti_2−x_ (PO_4_)_3_ (LATP)	^2^ LiClO_4_	0.52 (25 °C)	4.80	-	-	[75]
Li_1.3_Al_0.3_Ti_1.7_(PO_4_)_3_ (LATP)	LiTFSI LiTFSI	0.40 (25 °C) 7.47 (60 °C)	- 5.10	- -	- 152.8 (1 C)	[76] [77]
Li_1.5_Al_0.5_Ge_1.5_(PO_4_)_3_ (LAGP)	LiTFSI LiTFSI LiTFSI	1.67 (20 °C) 1.25 (25 °C) 0.44 (25 °C)	5.00 >3.8 5.10	- - -	- 143.6 (0.5 C) -	[78] [79] [80]
Li_1.4_Al_0.4_Ge_1.6_(PO_4_)_3_ (LAGP)	LiTFSI LiTFSI	1.72 (25 °C) 0.90 (30 °C)	- 5.12	- -	- 160.8 (50 °C)	[81] [82]
*Active Filler: Perovskite-Type*						
LSTZ	LiTFSI	0.54 (25 °C)	5.20	-	119	[83]
Li_0.35_La_0.55_TiO_3_ (LLTO)	LiTFSI	0.88 (25 °C)	5.10	-	-	[84]
Li_0.33_La_0.557_TiO_3_ (LLTO)	LiTFSI LiTFSI LiTFSI	1.30 (25 °C) 1.60 (25 °C) 2.40 (25 °C)	> 3.8 4.70 5.00	- - -	144.6 (60 °C, 1 C) 135 (60 °C, 2 C) -	[85] [86] [87]
Li_0.3_La_0.557_TiO_3_(LLTO)	LiTFSI LiTFSI	1.80 (25 °C) 2.30 (25 °C)	4.50 -	- -	- 384 (30 °C, 0.2 C)	[88] [89]
*Active Filler: Sulfide-Type*						
Li_6_PS_5_Cl (LPSC)	LiTFSI - LiTFSI	11.0 (25 °C) 10.0 (80 °C) 36.0 (80 °C)	4.90 >4.0 -	- - -	135.8 (0.05 C) 110.2 (60 °C) -	[90] [91] [92]
Li_6_PS_5_Cl/SiO_2_ (LPSC)	^2^ LiClO_4_	30.0 (25 °C)	> 4.2	-	134.3 (60 °C, 1 C)	[93]
Li_10_GeP_2_S_12_ (LGPS)	LiTFSI LiTFSI	1.80 (25 °C) 2.20 (25 °C)	>3.0 -	- -	588 (0.1 C) -	[94] [95]
Li_10_SnP_2_S_12_ (LSPS)	LiTFSI	1.70 (50 °C)	5.00	-	-	[96]

^1^ EW = electrochemical window. ^2^ LiClO_4_ = lithium perchlorate.

**Table 4:** Characterization techniques and their functionalities.

Characterization Techniques	Purpose
Fourier-transform infrared spectroscopy (FTIR)	Identify functional groups and chemical bonding
X-ray diffraction (XRD)	Determine crystal structure and phase composition
Differential scanning calorimetry (DSC)	Measure thermal properties and phase transitions
Scanning electron microscopy (SEM)	Observe morphology and surface, cross-sectional structure
Transmission electron microscopy (TEM)	Examine fine structural details
Nuclear magnetic resonance (NMR)	Study local molecular environment and dynamics
Electrochemical impedance spectroscopy (EIS)	Assess ionic conductivity and charge transport
Atomic force microscopy (AFM)	Analyze surface topology at the nanoscale
Raman spectroscopy	Probe vibrational modes and molecular interactions
Tensile tenting	Evaluate mechanical properties
X-ray photoelectron spectroscopy (XPS)	Analyze surface chemistry and elemental composition

## Data Availability

The original contributions presented in the study are included in the article, further inquiries can be directed to the corresponding author.
